# Design and Characterization of a Bioinspired PAMAM@Poly(Caffeic Acid) Magnetite Nanocarrier for Curcumin Delivery

**DOI:** 10.1021/acsomega.5c12312

**Published:** 2026-05-25

**Authors:** Alice Foti, Maria Kuznowicz, Bartosz F. Grześkowiak, Artur Jędrzak, Cristina Satriano, Teofil Jesionowski

**Affiliations:** † Nano Hybrid Biointerfaces Laboratory (NHBIL), Department of Chemical Sciences, University of Catania, Viale Andrea Doria 6, 95125 Catania, Italy; ‡ Interdisciplinary Centre for Ecotechnology, 49632 Poznan University of Technology, PL-61131 Poznan, Poland; § NanoBioMedical Centre, Adam Mickiewicz University in Poznan, Wszechnicy Piastowskiej 3, 61614 Poznan, Poland; ∥ Institute of Chemical Technology and Engineering, Faculty of Chemical Technology, 49632 Poznan University of Technology, Berdychowo 4, 60965 Poznan, Poland

## Abstract

Efficient intracellular drug delivery requires well-designed nanocarriers capable of stabilizing and releasing bioactive compounds in a controlled manner. Here, we introduce a bioinspired nanohybrid system that integrates generation 4.0 poly­(amidoamine) (PAMAM) dendrimers with poly­(caffeic acid)-coated magnetite nanoparticles (PCA@Fe_3_O_4_) to achieve efficient, long-lasting delivery of curcumin. Encapsulation within the PAMAM–PCA@Fe_3_O_4_ framework markedly improves curcumin stability and supports controlled release, with a total loading of approximately 190 μg of curcumin per sample as quantified by UV–Vis spectroscopy at 429 nm. AFM measurements showed progressive size increases from ∼26 nm for bare Fe_3_O_4_ to ∼43 nm for PCA@Fe_3_O_4_ and ∼66 nm for PAMAM–PCA@Fe_3_O_4_, while TEM analyses confirmed the spherical morphology of the nanoparticles. Release studies in PBS (pH 7.4, 37 °C) demonstrated a gradual and sustained release profile, with ∼33% of the encapsulated curcumin (∼64 μg) released over 24 h. In vitro cytotoxicity assays in MRC-5 fibroblasts indicated that PAMAM–PCA@Fe_3_O_4_ was biocompatible, whereas curcumin-loaded nanohybrids produced dose-dependent cytotoxicity consistent with curcumin’s known biological activity.

## Graphical abstract

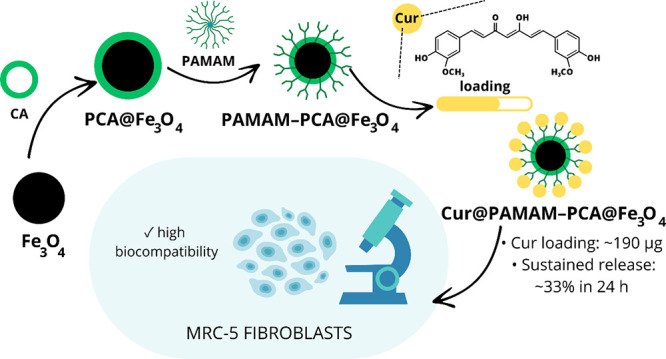

## Introduction

Recent advances in drug delivery have shifted the focus from conventional formulations toward multifunctional nanoscale systems capable of enhancing therapeutic efficacy and improving pharmacokinetic performance.^1^ Despite progress in molecular therapeutics, many clinically relevant compounds remain limited by poor solubility, rapid metabolic degradation, low bioavailability, and inefficient biodistribution.^2^ Nanotechnology offers a powerful platform to address these challenges by enabling site-specific, stimuli-responsive, and sustained delivery of bioactive agents.^3–6^ Nanocarriers can enhance the solubility and bioavailability of hydrophobic drugs, reduce systemic toxicity, protect labile compounds from premature degradation, and provide controlled release profiles that optimize therapeutic action.^7–11^

However, many currently available nanocarriers lack integrated functionality, often requiring additional targeting ligands or complex modifications to achieve selective and controlled delivery.

Poly­(amidoamine) (PAMAM) dendrimers, particularly generation 4.0, provide high drug loading capacity and facilitate cellular internalization through electrostatic interactions with negatively charged membranes.^12,13^ They offer a high density of surface amines for conjugation, enhancing drug stability and cellular uptake, and efficient delivery for biomedical applications.^14–16^ However, limitations of PAMAM-based systems include potential cytotoxicity associated with high surface amine density and insufficient structural stability without a supporting core. Moreover, dendrimers alone lack intrinsic magnetic guidance capability and redox-responsive behavior.

Superparamagnetic magnetite nanoparticles (Fe_3_O_4_) offer complementary advantages, including magnetic responsiveness, high surface-to-volume ratio, and established biocompatibility in biomedical applications such as MRI and targeted delivery.^17–24^ Magnetic nanoparticles enable external field-guided localization, and have shown promise in wound healing, especially when combined with natural compounds,^25^ yet unmodified Fe_3_O_4_ particles are prone to aggregation and surface oxidation, which can compromise colloidal stability and drug loading efficiency.

Surface engineering is therefore critical for achieving multifunctional behavior.

Poly­(caffeic acid) (PCA), a polymer derived from the natural antioxidant caffeic acid (CA), represents a bioinspired coating material rich in catechol groups and aromatic functionalities. Similar to other polyphenol-based coatings such as polydopamine,^26^ PCA can strongly adhere to inorganic surfaces while providing redox activity and abundant hydrogen-bonding sites, with a known capability of scavenging reactive oxygen species (ROS) and disrupting microbial membranes.^27–32^ Unlike inert coatings such as PEG, PCA offers inherent antioxidant, antibacterial, anti-inflammatory, and pH-responsive properties, which make it a promising multifunctional platform for wound healing applications.^33,34^ Its catechol groups enable π–π stacking, hydrogen bonding, and hydrophobic interactions with aromatic drugs such as curcumin, creating a chemically interactive microenvironment that enhances drug stabilization and retention.

Curcumin, a naturally occurring polyphenol derived from *Curcuma longa*, exhibits potent anti-inflammatory, antioxidant, and antimicrobial properties.^35^ It modulates cytokine expression, growth factor signaling, and oxidative stress responses, influencing all phases of wound healing from inflammation to remodeling.^36–39^ Nevertheless, its poor aqueous solubility and rapid metabolic degradation have limited its clinical utility.^39–43^

Here, we report a stepwise synthesis of Fe_3_O_4_ nanoparticles coated with PCA, functionalized with PAMAM dendrimers, and loaded with curcumin. The resulting Cur@PAMAM–PCA@Fe_3_O_4_ nanohybrid was characterized structurally and electrochemically, and evaluated for drug loading, release behavior, and cytocompatibility in human fibroblasts. The material qualifies as bioinspired because its catechol-mediated adhesion and molecular retention mimic polyphenol-based binding strategies observed in biological interfaces to achieve strong, selective interactions and controlled molecular retention.

While previous studies have explored PAMAM-functionalized magnetite systems or polymer-coated Fe_3_O_4_ nanoparticles independently, few have integrated a redox-active polyphenolic interlayer that simultaneously enhances drug affinity, antioxidant function, and interfacial stability. The present work addresses this gap through the rational design of a hierarchical hybrid nanocarrier composed of: (i) a magnetite (Fe_3_O_4_) core providing magnetic responsiveness and structural integrity; (ii) a poly­(caffeic acid) intermediate layer supplying redox activity, strong surface adhesion, and drug-interactive functionality; (iii) an outer G4 PAMAM dendrimer shell enabling high drug loading and favorable electrostatic interaction with cell membranes. The hypothesis beyond this design strategy is that integrating a catechol-rich polyphenolic interface between magnetite and PAMAM would enhance curcumin loading via synergistic π–π and hydrogen bonding interactions, stabilize the nanostructure against aggregation, modulate release kinetics through a diffusion–interaction coupled mechanism, and improve functional bioavailability through enhanced cellular association.

By combining magnetic responsiveness, dendrimer-mediated encapsulation, and bioactive polyphenolic coating, the platform aims to deliver curcumin in a stabilized, sustained, and multifunctional manner suitable for wound healing applications. The novelty of the proposed system lies in employing poly­(caffeic acid) multifunctional, intrinsically bioactive interfacial layer distinct from conventional PAMAM-coated or PEGylated magnetite-based nanocarriers, providing a chemically interactive and redox-responsive environment that enhances curcumin stabilization and delivery performance.

## Results

### Physicochemical Characterization

Comprehensive characterization was performed using UV–vis spectroscopy, FTIR, TEM, AFM, zeta potential analysis based on electrophoretic light scattering (ELS), noninvasive back-light scattering (NIBS), and cyclic voltammetry (CV). Zeta potential, hydrodynamic diameter, and polydispersity index (PdI) were measured at pH 7.0 to assess colloidal stability (Table 1).

**Table 1:** Zeta Potential, Hydrodynamic Diameter, and PdI of Synthesized Nanomaterials

sample	zeta potential (mV)	average size (nm)	PdI
Fe_3_O_4_	–46.3	123 ± 5	0.238
PCA@Fe_3_O_4_	–32.3	354 ± 6	0.745
PAMAM–PCA@Fe_3_O_4_	+15.2	411 ± 8	0.652

Unmodified Fe_3_O_4_ nanoparticles exhibited an average hydrodynamic diameter of 123 ± 5 nm with a PdI value of 0.238. A PdI below 0.3 indicates a relatively narrow size distribution and acceptable monodispersity, suggesting limited aggregation and good colloidal stability. The strongly negative zeta potential (−46.3 mV) further confirms effective electrostatic stabilization of the magnetic core.

After coating with poly­(caffeic acid), the average hydrodynamic diameter increased markedly to 354 ± 6 nm, accompanied by a substantial rise in PdI to 0.745. The increase in size reflects successful formation of a polymeric shell and enhanced hydration of the particle surface. The higher PdI value indicates a broader size distribution, which is typical for polymer-coated nanoparticles due to heterogeneous shell thickness and dynamic polymer chain conformations in aqueous media. Importantly, this increase does not necessarily imply irreversible aggregation but rather structural complexity introduced by the polymer layer. The simultaneous decrease in zeta potential to −32.3 mV confirms modification of the surface chemistry.

Subsequent PAMAM conjugation further increased the hydrodynamic diameter to 411 ± 8 nm and shifted the zeta potential to +15.2 mV, confirming dendrimer attachment. Although the PdI remained elevated (0.652), such values are commonly observed for multilayered polymeric nanostructures and reflect surface restructuring and increased interfacial heterogeneity. The absence of drastic size escalation beyond the expected shell thickness suggests preservation of colloidal integrity rather than uncontrolled particle aggregation.

Overall, the progressive increase in hydrodynamic diameter and systematic changes in PdI and zeta potential collectively confirm stepwise surface functionalization and formation of a multilayer core–shell nanostructure.

The results of physicochemical characterization by FTIR, cyclic voltammetry and XPS are presented in Figure 1. The FTIR spectra in the Figure 1A enable a direct comparison of the chemical structure of three samples: Fe_3_O_4_ nanoparticles, caffeic acid (CA), and poly­(caffeic acid)-coated magnetite (Fe_3_O_4_@PCA). The spectrum of pristine Fe_3_O_4_ exhibits a characteristic and intense absorption band at approximately 580 cm^–1^, corresponding to the Fe–O stretching vibrations typical for the spinel structure.^44^ The presence of this band confirms the integrity of the magnetic core following the surface modification steps.

**Figure 1:**
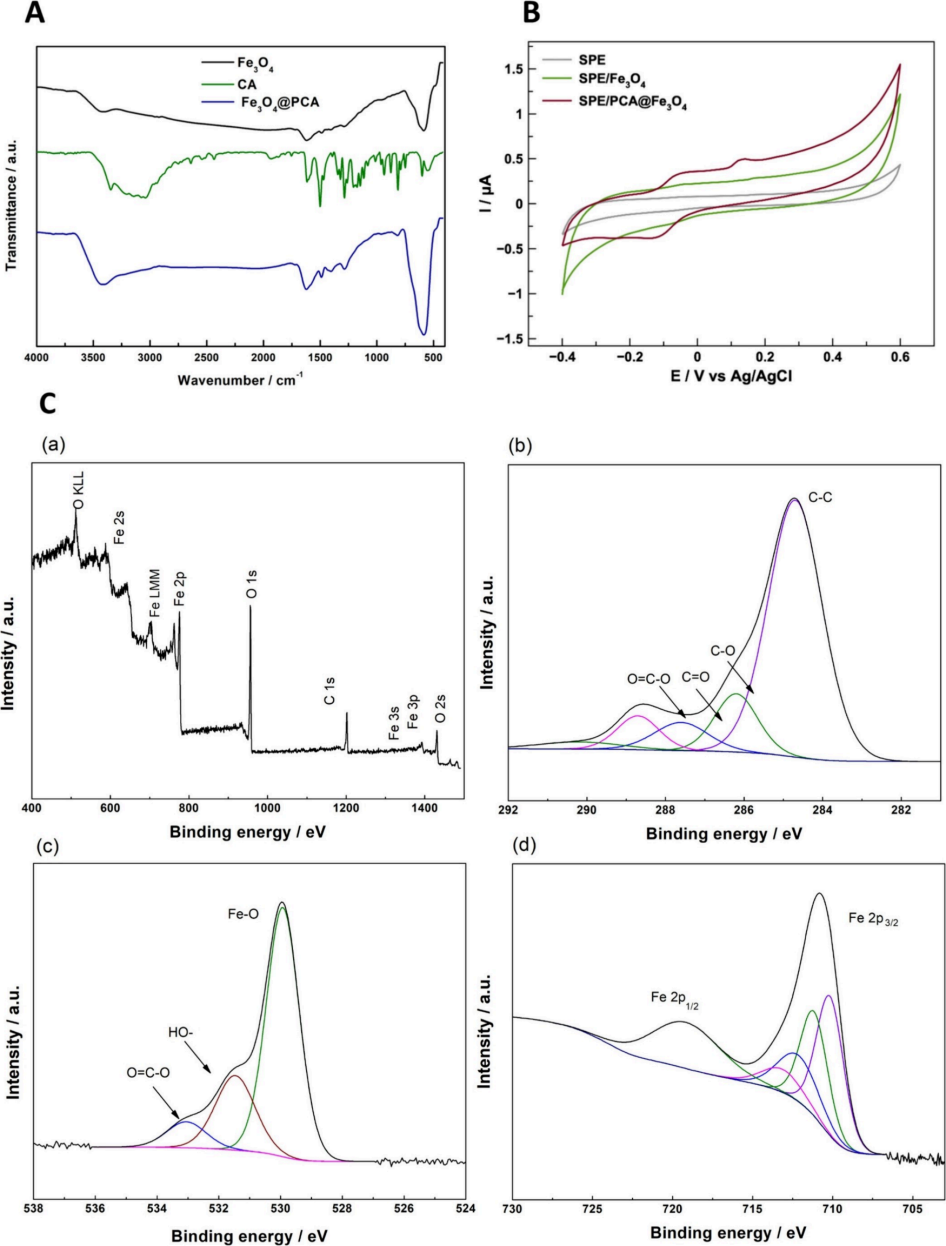
(A) FTIR spectra of Fe_3_O_4_, CA, and PCA@Fe_3_O_4_; (B) CV curves of bare SPE, SPE/Fe_3_O_4_, and SPE/PCA@Fe_3_O_4_ in PBS (50 mM, pH 7.4) at 10 mV s^–1^; (C) XPS spectrum of the hybrid Fe_3_O_4_@PCA (a); XPS results of C 1s (b); O 1s (c); Fe 2p (d).

The spectrum of caffeic acid displays several well-defined features associated with polyphenolic compounds. A broad absorption band in the range of 3200–3500 cm^–1^ is attributed to O–H stretching vibrations of phenolic and carboxylic groups. The strong band near 1680–1700 cm^–1^ corresponds to the C=O stretching of the carboxyl group. Additional peaks at around 1600 and 1510 cm^–1^ originate from aromatic C=C stretching, while the bands in the 1050–1250 cm^–1^ region are assigned to C–O stretching in phenolic and alcoholic groups.^45^

The FTIR spectrum of Fe_3_O_4_@PCA combines the features of both the inorganic core and the organic poly­(caffeic acid) coating. The Fe–O band at approximately 580 cm^–1^ remains present, indicating that the magnetite structure is preserved. At the same time, the broadening and increased intensity of the O–H stretching region (3200–3500 cm^–1^) suggest enhanced hydrogen bonding and interactions between PCA hydroxyl groups and the nanoparticle surface. The attenuation of the C=O band relative to free CA implies involvement of the carboxyl group in chemisorption onto Fe_3_O_4_. Furthermore, the presence of bands in the 1050–1150 cm^–1^ region corresponds to C–O stretching in catechol-derived structures, confirming successful deposition of the polyphenolic coating.

Overall, the observed shifts and intensity changes in the Fe_3_O_4_@PCA spectrum provide strong evidence of effective surface functionalization with poly­(caffeic acid). The FTIR data verify the formation of a stable catechol-based coating, consistent with the expected binding behavior of polyphenols on oxide surfaces.

CV analysis (Figure 1B) provided a more definitive confirmation of the efficiency of the synthesis and the successful coating of PCA onto the Fe_3_O_4_ surface. These electroactive signals, which are absent in unmodified Fe_3_O_4_, directly indicate the presence of surface-bound catechol groups.

The stability of the redox responses over successive scans further supports the formation of a robust poly­(caffeic acid) layer rather than weak physisorption.^46,47^

XPS analysis (Figure 1C) was carried out to determine the elemental composition and oxidation states of the material. The survey spectrum recorded for Fe_3_O_4_@PCA (Figure 1C, panel a) confirmed the presence of carbon, oxygen, and iron, which are expected components of the final structure. The deconvoluted C 1s spectrum (Figure 1C, panel b) displayed signals at 284.4, 286.1, 287.3, and 288.4 eV, corresponding to C–C, C–O, C=O, and O=C–O functionalities, respectively. In the O 1s region (Figure 1C, panel c), three contributions were detected at 529.9, 530.7, and 531.1 eV, associated with Fe–O bonds, hydroxyl groups, and O=C–O moieties. The Fe 2p spectrum (Figure 1C, panel d) exhibited peaks at 711.2 eV (Fe 2p__3_/_2__) and 720.4 eV (Fe 2p__1_/_2__), confirming that the magnetite surface was successfully modified with poly­(caffeic acid).

### Drug Loading and Release

Curcumin was encapsulated into PAMAM–PCA@Fe_3_O_4_ with ∼95% efficiency (corresponding to ∼190 μg of curcumin), as quantified by UV–vis at 429 nm. Release studies in PBS (pH 7.4, 37 °C) demonstrated a sustained and gradual release profile, with ∼33% of curcumin (∼64 μg) released over 24 h. This controlled release behavior indicates the potential of the nanocarrier system for prolonged therapeutic action, enabling continuous bioavailability of curcumin at the wound site (Figure 2).

**Figure 2:**
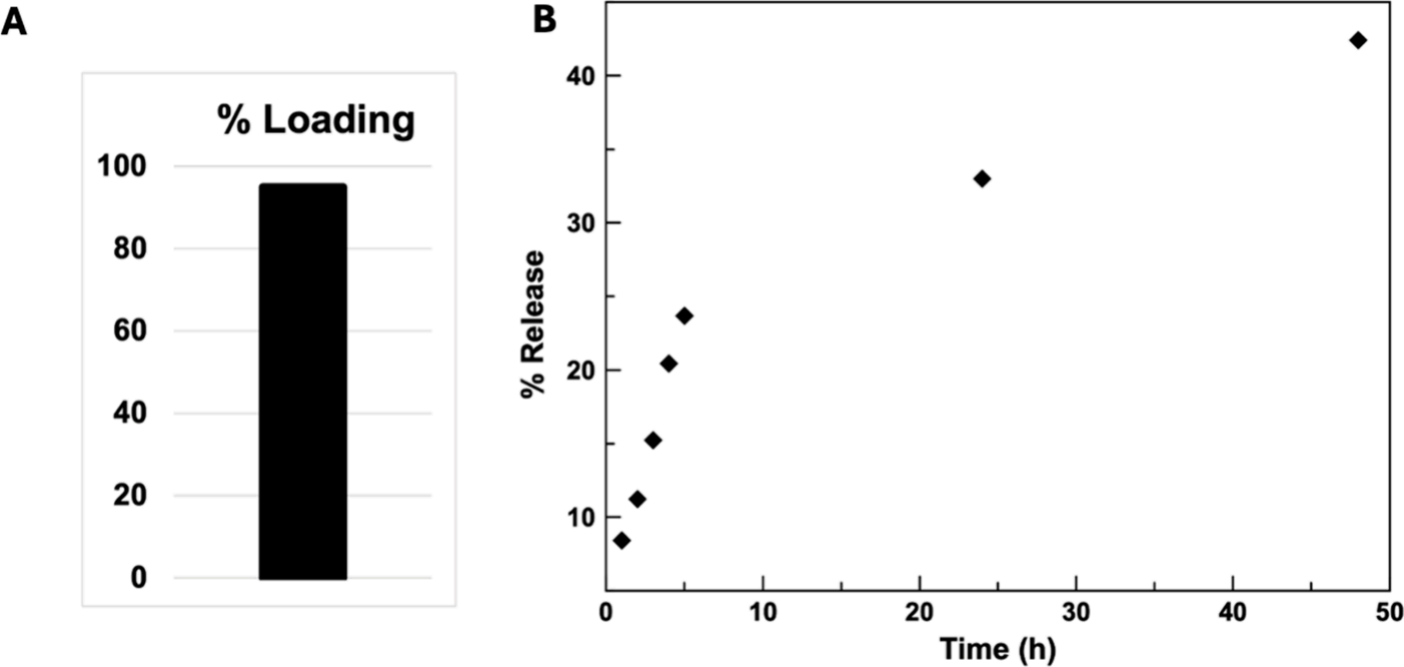
(A) Curcumin loading efficiency; (B) cumulative release profile over 48 h.

The release profile of curcumin from the PAMAM–PCA@Fe_3_O_4_ nanocarrier exhibits a characteristic biphasic pattern typical of multilayer polymeric systems. The high encapsulation efficiency (∼95%) confirms strong interactions between curcumin and the functional groups present in both the PCA coating and the PAMAM dendrimer layer. Hydrogen bonding and π–π interactions between aromatic moieties contribute to effective stabilization of curcumin within the carrier matrix, limiting premature desorption.

An initial mild burst release of approximately 10% is observed during the first hours of incubation. This fraction likely corresponds to curcumin molecules weakly bound or located near the outer surface of the nanocarrier. After this early phase, the system transitions into a sustained and gradual release stage that continues over 48 h, reaching approximately 43% cumulative release. The absence of any abrupt increase at later time points indicates structural stability of the polymeric layers under the experimental conditions.^48,49^

To elucidate the release mechanism, the experimental data were analyzed using classical kinetic models. The release curve shows good agreement with the Higuchi model, suggesting diffusion-controlled transport from a polymeric matrix. Further evaluation using the Korsmeyer–Peppas equation yielded a diffusion exponent *n* < 0.5, confirming that the release process follows Fickian diffusion. These findings indicate that curcumin transport is governed primarily by molecular diffusion through the PCA and PAMAM layers rather than by carrier erosion or degradation.^46,47^

Mechanistically, drug release results from the interplay between concentration-gradient-driven diffusion and limited polymer chain relaxation. Because no significant degradation of the carrier occurs during the experiment, the release rate remains controlled and progressive. The combination of high loading efficiency, minimal burst release, and diffusion-dominated kinetics demonstrates that PAMAM–PCA@Fe_3_O_4_ functions as a stable and effective controlled-release platform for curcumin.

In addition to encapsulation efficiency (EE), drug loading capacity (LC) was considered as a key parameter describing formulation performance. The high EE obtained in this system reflects the strong affinity of curcumin for the multifunctional polymeric interface and indicates efficient utilization of the carrier surface, consistent with values reported for highly functionalized dendrimer-based nanocarriers in the literature.

Beyond these quantitative indicators, structural and physicochemical criteria are essential for a meaningful comparison of loading performance among different studies. These include potential alterations in particle size distribution, surface charge characteristics, and colloidal stability following drug incorporation. Although these specific values were not part of the data set provided, high loading often correlates with minimal disruption of the polymeric shell architecture, particularly in dendrimer-based platforms where internal cavities and abundant terminal groups facilitate drug entrapment without inducing aggregation.

A further important criterion involves the interaction profile of curcumin within the nanocarrier, which affects both loading efficiency and subsequent release behavior. In the presented system, the stability of curcumin within the polymer matrix can be inferred from the moderate initial burst release and the sustained release pattern observed over 48 h. This slow and diffusion-dominated release is consistent with strong internal retention during loading, confirming that the majority of curcumin molecules occupy deeper regions of the PAMAM–PCA@Fe_3_O_4_ layers rather than remaining loosely bound at the surface.

AFM analysis provided detailed insight into the morphological evolution of the nanostructures at each stage of functionalization and drug loading (Figure 3). The unmodified Fe_3_O_4_ nanoparticles (Figure 3A) showed a comparatively smooth surface with minimal height fluctuations reaching approximately 26 nm. This uniform topography is consistent with the compact, weakly aggregated character expected for magnetite nanoparticles stabilized in the absence of additional surface modifiers. Deposition of the poly­(caffeic acid) layer (PCA@Fe_3_O_4_, Figure 3B) resulted in a distinct increase in surface roughness and heterogeneity. The enhancement of the vertical profile to around 43 nm suggests the formation of an adherent polymer coating, which introduces nanoscale irregularities associated with the amorphous nature of PCA. The broadened topographical features imply an initial development of a more complex interfacial architecture capable of supporting subsequent chemical modifications. Following PAMAM dendrimer grafting (PAMAM–PCA@Fe_3_O_4_, Figure 3C), the surface underwent a pronounced morphological transformation. The emergence of densely packed, irregular protrusions and the expansion of maximum height to approximately 66 nm reflect the characteristic branched structure of PAMAM and its tendency to assemble nonuniformly on textured polymer layers. This increase in structural volume indicates successful conjugation and suggests enhanced functional-group availability, which is desirable for drug binding and further biomolecular interactions. Complementary TEM micrographs (Figure 3E,F) further verify the spherical morphology of the nanoparticles. The final curcumin-loaded system (Cur@PAMAM–PCA@Fe_3_O_4_, Figure 3D) exhibited the most complex and voluminous surface architecture among all samples.

**Figure 3:**
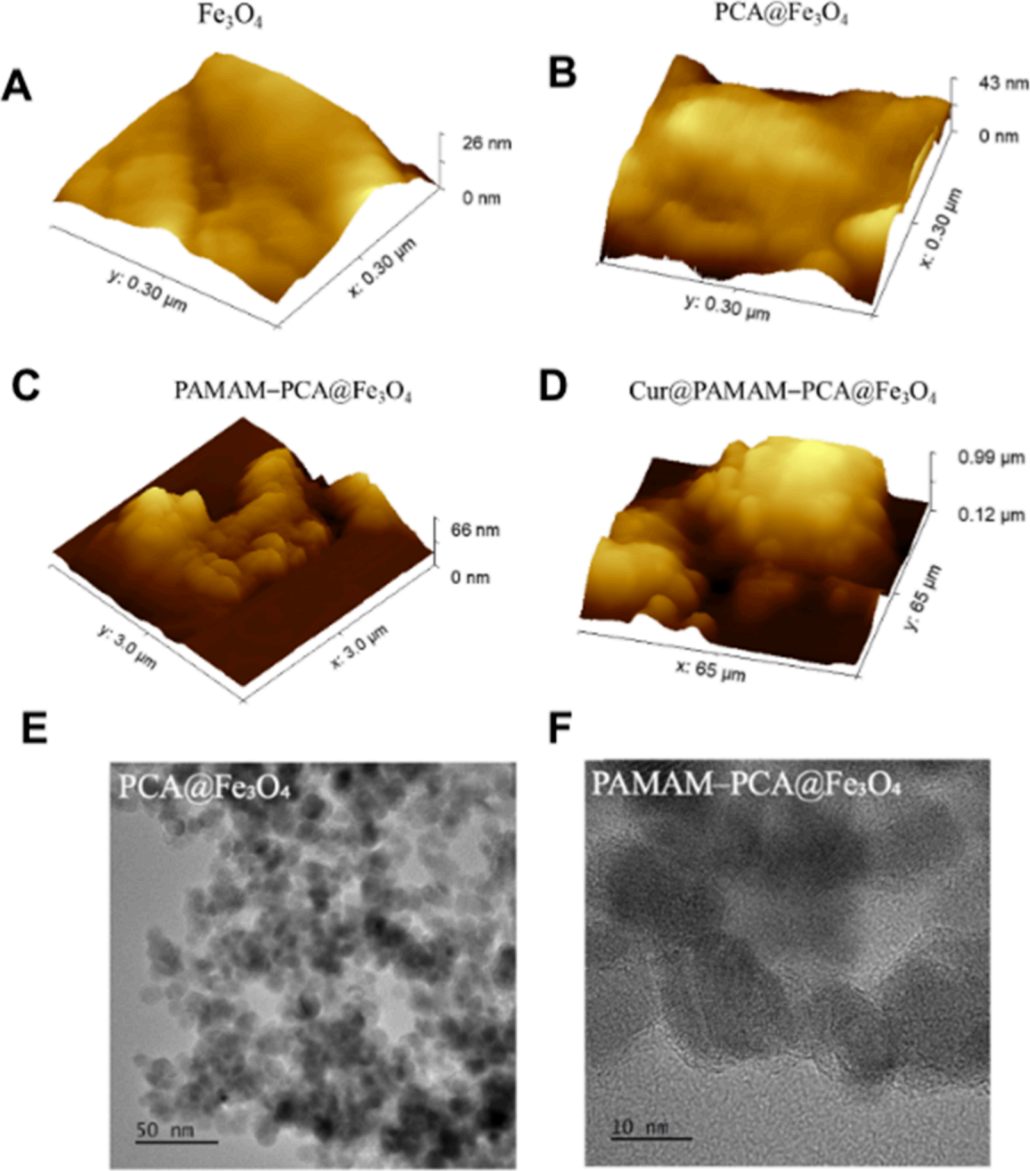
AFM images of (A) Fe_3_O_4_; (B) PCA@Fe_3_O_4_; (C) PAMAM–PCA@Fe_3_O_4_; and (D) Cur@PAMAM–PCA@Fe_3_O_4_; TEM micrographs of (E) PCA@Fe_3_O_4_ and (F) PAMAM–PCA@Fe_3_O_4_.

The topography was dominated by prominent, globular aggregates and a dramatic increase in vertical dimensions, nearing 1 μm within localized regions. This pronounced growth in surface features is attributable to incorporation of the hydrophobic drug, which can promote localized clustering of the polymer–dendrimer matrix. The resulting morphology is characteristic of drug-loaded hybrid nanocarriers where the therapeutic cargo influences the packing, hydration, and swelling behavior of the soft organic shell.^50,51^

### Cell Viability Assays

WST-1 assays in MRC-5 fibroblasts indicated that PAMAM–PCA@Fe_3_O_4_ was biocompatible, while curcumin-loaded nanohybrids exhibited dose-dependent cytotoxicity (Figure 4A), consistent with curcumin’s known pro-apoptotic activity.^52^ Notably, treatment with free curcumin at equivalent concentrations resulted in minimal biological effect, highlighting the improved functional delivery and bioavailability achieved when curcumin is associated with the nanocarrier.

**Figure 4:**
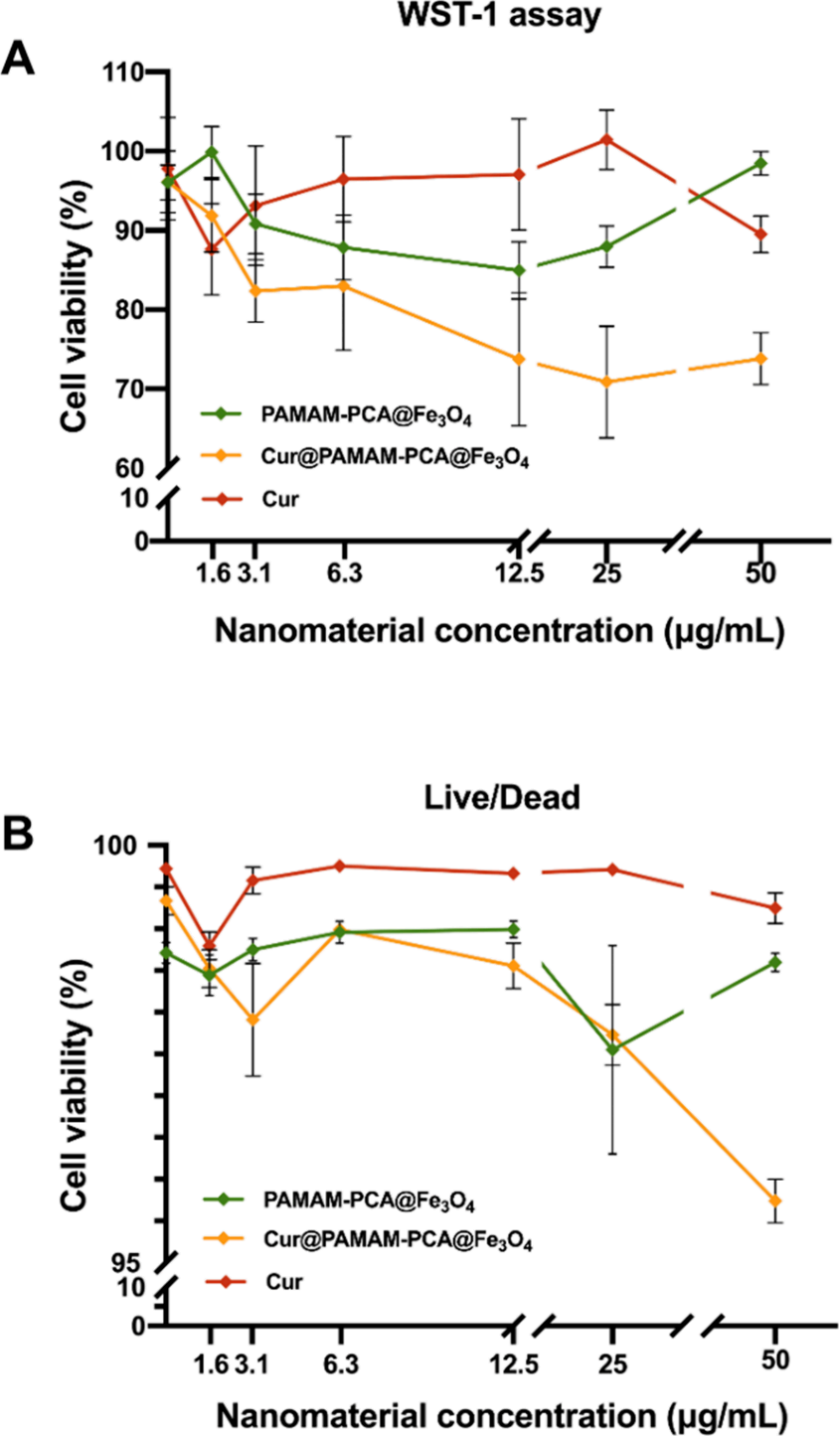
Cell viability of MRC-5 fibroblasts after exposure (48 h) to PAMAM–PCA@Fe_3_O_4_, Cur@PAMAM–PCA@Fe_3_O_4_, and free curcumin, as assessed by quantitative analyses of WST-1 (A) and fluorescence live/dead (B) assays. Data are presented as mean ± standard deviation (SD).

Live/dead imaging corroborated these results (Figure 4B and 5). Cells treated with PAMAM–PCA@Fe_3_O_4_ (Figure 5A) maintained high viability, whereas Cur@PAMAM–PCA@Fe_3_O_4_ (Figure 5B) induced increased death. Free curcumin (Figure 6C) had negligible effects. Controls confirmed assay validity (Figure 5D,E).

**Figure 5:**
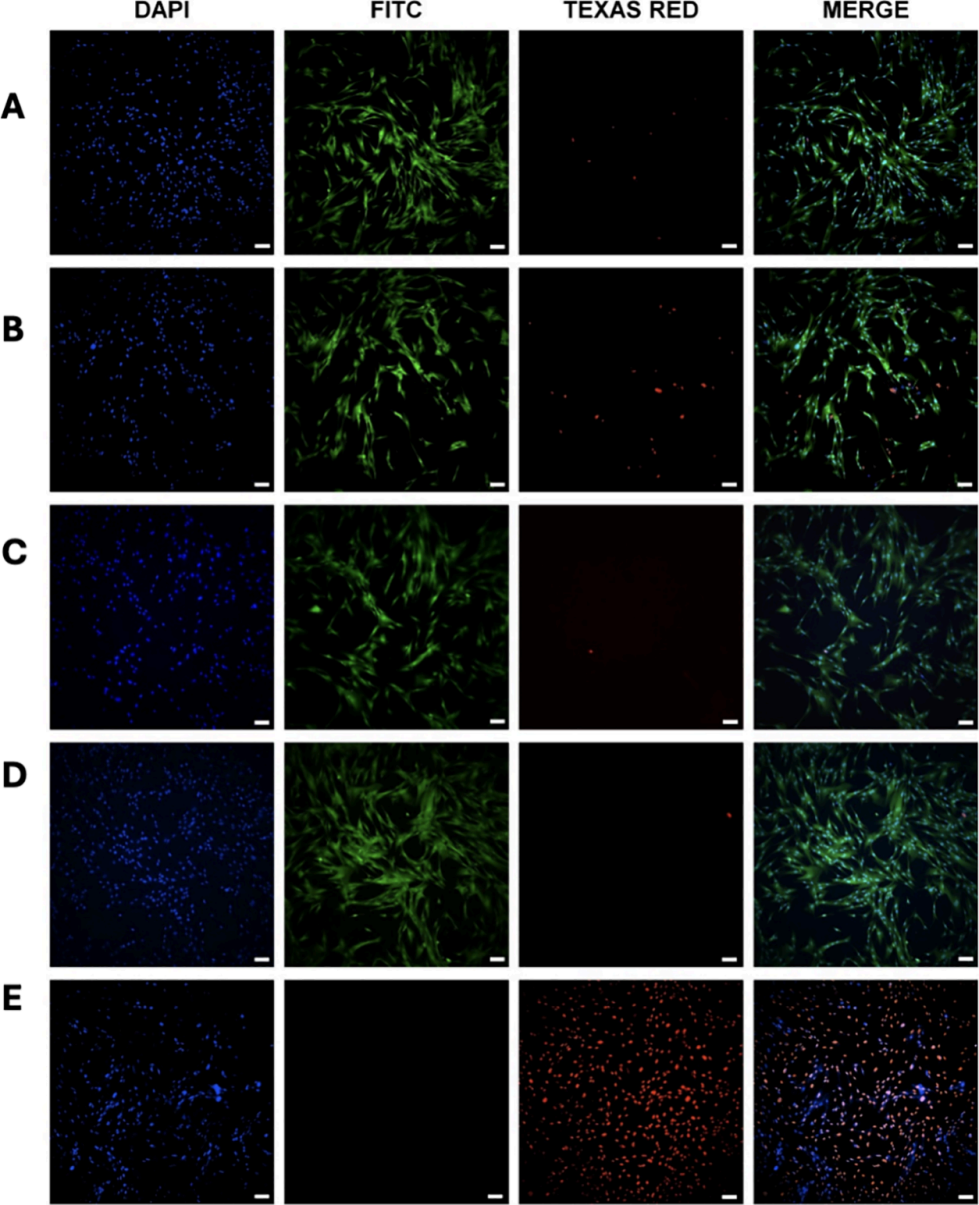
Live/dead fluorescence images of MRC-5 cells exposed to (A) PAMAM–PCA@Fe_3_O_4_, (B) Cur@PAMAM–PCA@Fe_3_O_4_, (C) free curcumin, (D) untreated, and (E) DMSO-treated controls. Scale bar = 100 μm. Cell seeding was performed using standardized pipetting and mixing procedures, ensuring uniform distribution. The micrograph reflects typical fibroblast morphology and spatial distribution.

**Figure 6:**
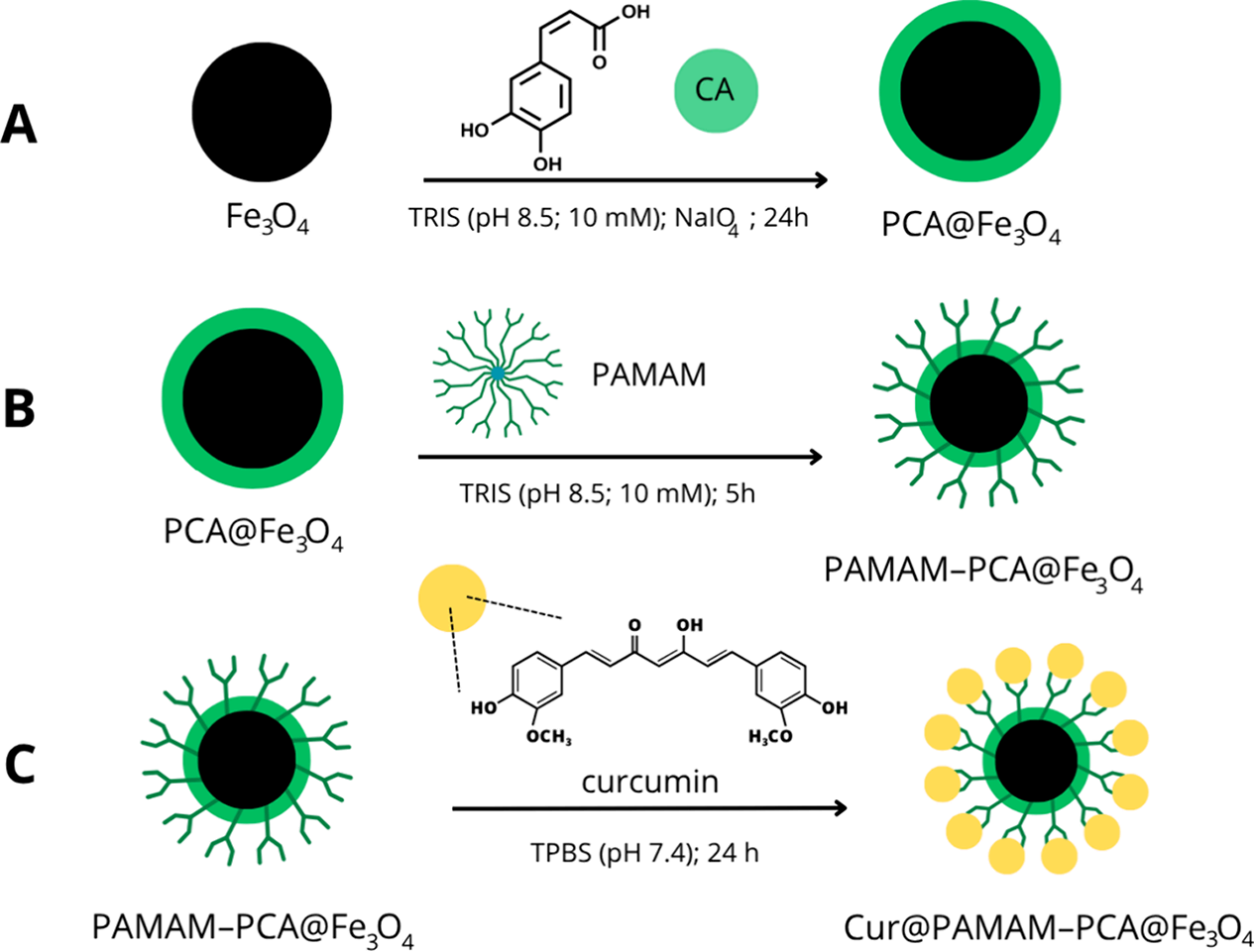
Schematic representation of the individual synthesis steps: polymerization of magnetite nanoparticles with poly­(caffeic acid) (A); synthesis of PAMAM–PCA@Fe_3_O_4_ (B); loading of curcumin (Cur) (C).

## Discussion

Previously reported PAMAM–Fe_3_O_4_ nanocarriers typically enhance performance via direct dendrimer functionalization, PEGylation, or ligand/antibody conjugation, yielding improvements in biocompatibility, pH-responsive release, or molecular recognition but generally along a single mechanistic axis. For instance, PEG-modified PAMAM–Fe_3_O_4_–DOX triads exhibited generation-dependent increases in drug loading with preferential drug release at acidic pH, underscoring the efficacy of polymeric surface engineering for endosomal environments.^53^ Polydopamine coated Fe_3_O_4_ modified with PAMAM showed nucleic-acid condensation and transfection through catechol-enabled adhesion and passivation, yet they mainly contribute recognition rather than interfacial redox functionality without addressing sustained antioxidant protection for polyphenolic drugs.^54^

In contrast, our Cur@PAMAM–PCA@Fe_3_O_4_ nanoconstruct introduces a catechol-rich poly­(caffeic acid) interlayer that furnishes intrinsic redox activity and a chemically interactive, polyphenolic interface between magnetite and the dendrimer. Catechol/quinone pairs within poly­(catechol) coatings accelerate Fe^3+^ → Fe^2+^ regeneration and broaden operational pH windows by leveraging reversible phenolic/quinonoid redox cycling, while simultaneously improving dispersity and rendering more negative surface charge, mechanistic features directly observed on Fe_3_O_4_ modified with poly­(catechol) and transferrable to PCA scaffolds. Polyphenol interfaces also enable multiple noncovalent and covalent interactions (H-bonding, π–π stacking, Michael/Schiff reactions, and metal coordination), which are particularly well-matched to curcumin’s aromatic and phenolic structure, thereby stabilizing payloads and strengthening drug–matrix cooperativity beyond what PEGylation or antibody ligation alone provide. Moreover, PCA coatings are established as bioactive, catechol-based films capable of deposition under mild conditions and contributing antioxidant and antibacterial functionality, further supporting their role as a redox-active interlayer in biomedical hybrids. Collectively, the hierarchical Fe_3_O_4_ core → PCA interlayer → PAMAM shell consolidates magnetic guidance, polyphenol-mediated redox stabilization/interactive binding, and high-capacity dendrimer encapsulation within a *single* architecture—an integration not simultaneously realized in earlier PAMAM–magnetite systems that typically deploy only one or two of these mechanisms in isolation.

The stepwise surface modification of Fe_3_O_4_ was validated through electrokinetic, spectroscopic, and electrochemical techniques.

The progressive shift in zeta potential from −46.3 mV (bare Fe_3_O_4_) to −32.3 mV after PCA coating, and finally to +15.2 mV following PAMAM functionalization, demonstrates successful multilayer assembly and surface charge reversal. The positive surface charge reflects the presence of protonated amine groups from G4 PAMAM, which are known to enhance electrostatic interaction with negatively charged cellular membranes.^55^

The concurrent increases in hydrodynamic diameter and polydispersity index further support multilayer formation. While moderate aggregation was observed, the PdI values remained within an acceptable range for polymer-coated magnetic systems, indicating colloidal stability.

FTIR, XPS and cyclic voltammetry analyses confirmed successful immobilization of PCA via catechol-surface coordination.^56^ Characteristic redox peaks corresponding to catechol/quinone transitions, provided direct evidence for the retention of electroactive polyphenolic functionality, consistent with previous reports on catechol-based surface coatings.^57,58^ The preservation of redox activity is particularly relevant for antioxidant performance and microenvironmental modulation in wound-healing contexts.

The PAMAM–PCA@Fe_3_O_4_ nanocarrier exhibited high curcumin encapsulation efficiency (∼95%). This excellent loading capacity can be attributed to synergic interactions including: (i) strong hydrophobic within PAMAM internal cavities, (ii) π–π stacking between curcumin’s aromatic rings and PCA’s phenolic structure; and (iii) hydrogen bonding between curcumin’s hydroxyl groups and catechol moieties. Morphological changes observed via AFM and TEM indicated successful drug incorporation and preservation of core–shell structure. The core diameter (10–20 nm) and surface reorganization following curcumin loading are consistent with efficient hybrid formation and drug–matrix interaction.

The sustained release profile (∼33% over 24 h) under physiological conditions supports its utility for prolonged drug delivery in wound care. Kinetic modeling (Korsmeyer–Peppas fit of diffusion exponent *n* < 0.5) suggests that the drug release mechanism is primarily governed by quasi-Fickian diffusion, consistently with diffusion-controlled transport (as indicated by the Higuchi model) and cooperative contribution of dendrimer encapsulation and polyphenolic binding, which hinders the rapid diffusion of the molecule. Thus, physicochemical architecture directly influences biological performance by stabilizing curcumin while permitting controlled release.

Cyclodextrins, particularly β-CD and its derivatives, are widely used as curcumin carriers due to the simplicity of inclusion complex formation and good biocompatibility. However, the curcumin binding mechanism in cyclodextrins is primarily based on hydrophobic interactions within a single cavity, which limits maximum loading and the ability to control release kinetics. In contrast, PAMAM dendrimers offer a three-dimensional, highly branched architecture with a large number of functional groups, enabling the simultaneous binding of multiple curcumin molecules both in the core and on the dendrimer surface. This results in significantly higher loading capacity and greater encapsulation efficiency compared to cyclodextrins.^59–61^

Biocompatibility assays confirmed minimal toxicity of the unloaded nanocarrier, demonstrating that PCA coating and PAMAM conjugation effectively mitigate potential surface reactivity of bare Fe_3_O_4_ nanoparticles. Literature indicates that catechol-containing coatings can reduce oxidative surface activity of magnetite by partial surface passivation.^62^ It should be clarified that the nanoparticles developed in this study were not functionalized with specific targeting ligands and were not designed for cell-type-specific recognition. The MRC-5 fibroblast line was used solely as a representative cellular model system to assess cytocompatibility and functional bioavailability. Although confocal microscopy and flow-cytometry quantification were not included in this work, there are evidence that support enhanced fibroblast-associated delivery of curcumin by the nanocarrier.

The enhanced cytotoxicity observed for Cur@PAMAM–PCA@Fe_3_O_4_ compared to free curcumin likely results from improved solubilization of curcumin in aqueous media, increased cellular uptake facilitated by the positively charged PAMAM shell, and sustained intracellular release. The dose-dependent cytotoxicity observed for Cur@PAMAM–PCA@Fe_3_O_4_ correlates with curcumin’s established pro-apoptotic activity, which is mediated through pathways such as ROS generation, mitochondrial membrane potential disruption, and caspase activation.^63^ In the wound-healing context, such controlled cytotoxicity toward overproliferative fibroblasts could be advantageous for preventing hypertrophic scar formation and modulating excessive extracellular matrix deposition, thereby promoting more balanced tissue regeneration. Thus, the observed dose-dependent response in fibroblasts indicates improved functional delivery rather than intrinsic nanocarrier toxicity.

These results position Cur@PAMAM–PCA@Fe_3_O_4_ as a multifunctional nanoplatform offering drug delivery, antioxidant capability, and tailored bioactivity. Despite promising results, several limitations must be acknowledged, as this study did not include ligand-mediated targeting studies to validate target specificity, long-term intracellular trafficking and degradation pathways, in vivo pharmacokinetics and biodistribution, and potential oxidative effects of iron-core nanoparticles under chronic exposure. Future studies should include advanced cellular uptake quantification (confocal microscopy and flow cytometry), hemocompatibility assessment, ROS profiling under prolonged exposure, and *in vivo* wound-healing models to establish translational potential.

## Conclusion

We report the design and comprehensive physicochemical and biological evaluation of a multifunctional nanocarrier, PAMAM–PCA@Fe_3_O_4_, for curcumin delivery in wound healing applications. The nanoplatform integrates magnetic responsiveness, redox-active polyphenolic coating, and dendrimer-mediated drug encapsulation. By directly linking structural architecture to loading efficiency, release kinetics and biological performance, this work demonstrates how rational interface engineering can transform individually useful materials into a synergistic, bioinspired therapeutic system.

Overall, the combined structural, electrochemical, and biological data consistently validate PAMAM–PCA@Fe_3_O_4_ as a promising and rationally engineered platform for topical drug delivery in regenerative medicine. Additional evaluations, such as magnetic hyperthermia and antibacterial testing, would further broaden the functional profile of this system but lie beyond the scope of the present study, which focuses on drug-delivery behavior and mechanistic characterization. Future work will therefore include expanded cytocompatibility studies, antibacterial assessment, long-term stability testing, hemocompatibility assessment, and mechanistic studies addressing oxidative stress pathways and *in vivo* validation to fully explore the therapeutic potential of this nanocarrier.

## Methods

### Chemicals

All reagents, including FeCl_3_·6H_2_O, FeCl_2_·4H_2_O, NH_4_OH (25%), citric acid, caffeic acid, sodium periodate, PAMAM dendrimer generation 4.0, curcumin, PBS (10 mM, pH 7.4), and TRIS buffer (10 mM, pH 8.5), were of analytical grade and purchased from Merck (Poland). Materials were used without further purification.

### Instrumentation

UV–visible (UV–Vis) spectra were recorded on a PerkinElmer Lambda 950 UV/vis/NIR spectrophotometer over the range of 200–900 nm. Fourier-transform infrared (FTIR) measurements were carried out using a Bruker Vertex 70 spectrometer with samples prepared as KBr pellets.

X-ray photoelectron spectroscopy (XPS) analysis was performed using using a PHI 5000 VersaProbe II instrument equipped with a monochromatic Al Kα X-ray source (1486.6 eV). High-resolution spectra of Fe 2p, C 1s, O 1s, and other relevant elements were acquired at a pass energy of 23.5 eV with an analyzer step size of 0.2 eV. Spectral fitting was carried out using the PHI processing software applying Shirley background correction. All binding energies were referenced to the adventitious carbon C 1s peak at 284.6 eV. The uncertainty associated with reported binding energy values is ±0.2 eV.

Atomic force microscopy (AFM) was conducted on an Agilent 5500 instrument operated in intermittent contact mode. Transmission electron microscopy (TEM) images were obtained using a JEOL JEM-1400 microscope operating at 120 kV with a resolution of 2 nm.

Electrochemical characterization by cyclic voltammetry (CV) was performed using a μ-Autolab III potentiostat (ECO Chemie, Netherlands) with Metrohm DropSens screen-printed electrodes at a scan rate of 10 mV·s^–1^ in phosphate-buffered saline (PBS, pH 7.4).

Zeta potential and nanoparticle size analysis were carried out using a Malvern Zetasizer Nano ZS employing electrophoretic light scattering (ELS) and noninvasive backscatter (NIBS) methods.

### Synthesis of PCA@Fe_3_O_4_

Magnetite nanoparticles (Fe_3_O_4_) were prepared via a chemical coprecipitation method. In this process, 3 g (11.09 mM) of iron­(III) chloride hexahydrate and 1.5 g (7.55 mM) of iron­(II) chloride tetrahydrate were dissolved in 100 mL of Milli-Q water. The mixture was heated to 90 °C, followed by the gradual addition of 20 mL of 25% aqueous ammonia. The reaction was conducted under a nitrogen atmosphere to minimize oxygen contact, thereby preserving the oxide state of magnetite. Upon formation of the iron oxide precipitate, the resulting black suspension was cooled to ambient temperature.

To synthesize magnetite@poly­(caffeic acid) nanoparticles (Fe_3_O_4_@PCA), 10 mg of previously prepared magnetite nanoparticles were dispersed in 20 mL of TRIS buffer solution (10 mM; pH 8.5). Subsequently, caffeic acid monomer (10 mg), in an amount equal to the Fe_3_O_4_ mass, was added. Sodium periodate (NaIO_4_) was introduced into the reaction system as an oxidizing agent to initiate and facilitate the polymerization of the monomer. The mixture. was stirred magnetically for 24 h at room temperature. This process is schematically presented in Figure 6A.

The resulting Fe_3_O_4_@PCA nanoparticles were then separated using a magnet, washed three times, and redispersed in Milli-Q water. The suspension was stored under refrigeration until further use. The process of polymerization was carried out chemically. Figure 7 shows a schematic illustration of this process.

**Figure 7:**
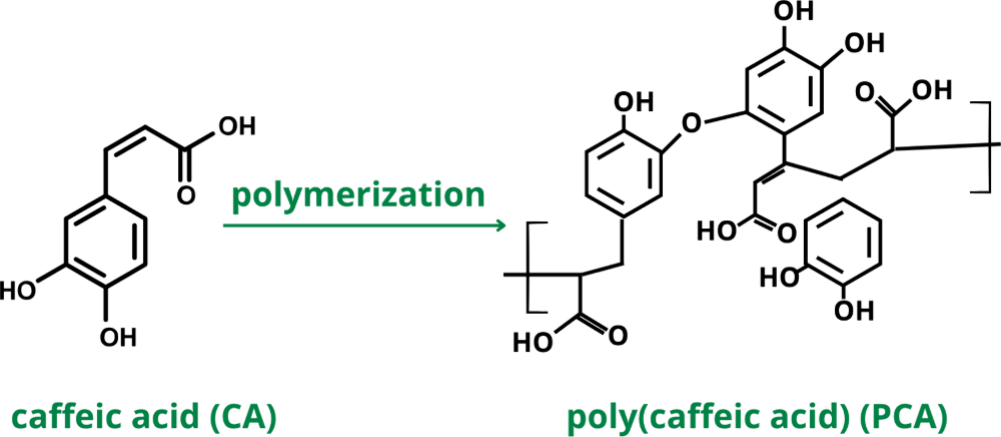
Polymerization scheme of the biopolymer—poly­(caffeic acid).

### Synthesis of PAMAM–PCA@Fe_3_O_4_

To fabricate dendrimer-grafted poly­(caffeic acid)@magnetite nanohybrids (PAMAM–PCA@Fe_3_O_4_), PCA@Fe_3_O_4_ nanoparticles (10 mg) were dispersed in TRIS buffer (10 mM, pH 8.5) containing 2.5 mg of PAMAM dendrimers (generation 4.0) and incubated under continuous magnetic stirring (600 rpm) for 5 h at ambient temperature.

The surface functionalization proceeded primarily via electrostatic interactions between the negatively charged poly­(caffeic acid) layer and the protonated amine groups of PAMAM, supported by hydrogen-bonding interactions.

Following incubation, the functionalized nanoparticles were magnetically separated and thoroughly washed with an ethanol/water mixture (1:1, v/v) to remove unbound dendrimers and residual reagents (Figure 6B).

### Curcumin Loading and Release

Curcumin (0.2 mg in acetone) was added to a suspension of PAMAM–PCA@Fe_3_O_4_ (1 mg in PBS), and the mixture was stirred for 24 h at ambient temperature to allow for adsorption and encapsulation (Figure 6C). Acetone was evaporated. Encapsulation was quantified by UV–vis at 429 nm.^64^ For release studies, 1 mg Cur@PAMAM–PCA@Fe_3_O_4_ was incubated in PBS (37 °C). Aliquots were collected at predetermined intervals and analyzed spectrophotometrically to monitor the release profile of curcumin.

### Cell Viability Assays

MRC-5 fibroblasts (ATCC) were cultured in DMEM + 10% fetal bovine serum (FBS). *WST-1 assay:* cells (3000/well, 96-well plates) were treated with 1.6–50 μg/mL nanoparticles for 24 h. All experiments were conducted using formulations normalized to the Fe_3_O_4_ content of the nanocarrier, with corresponding amounts of PAMAM and curcumin inherently determined by the synthesis stoichiometry. This ensures consistent comparison across all tested samples. Following treatment, cell viability was assessed by measuring absorbance at 450 nm with a reference wavelength of 620 nm using an Anthos Zenyth 340 rt Biochrom plate reader. *Live/Dead assay: c*ells were stained with Hoechst 33342, calcein AM, and ethidium homodimer-1 after 24 h nanoparticle exposure. Imaging was done with IN Cell Analyzer 2000 using DAPI, FITC, and Texas Red channels. At least 20 random fields per well were captured and analyzed to ensure statistical robustness. Data are presented as mean ± standard deviation (SD). To distinguish the effects of individual formulation components, appropriate control groups were included. In the WST-1 proliferation assay, reference groups comprised unloaded nanoparticles and free curcumin. All experimental results were normalized to untreated control cells that were not exposed to any material. In the live/dead assay, untreated cells served as the negative control, while DMSO-treated cells were used as the positive control.
